# Effect of aging on semen and embryonic developmental scores in assisted reproductive technology

**DOI:** 10.1002/rmb2.12647

**Published:** 2025-05-21

**Authors:** Taiyo Yamamoto, Katsuya Mine, Hisataka Iwata

**Affiliations:** ^1^ Department of Animal Science, Graduate School of Agriculture Tokyo University of Agriculture Atsugi City Kanagawa Japan

**Keywords:** developmental kinetics, female aging, iDAScore, male aging, time‐lapse

## Abstract

**Purpose:**

The effects of female aging on fertility have been extensively studied; however, this is not the case for aging males. Embryonic selection using time‐lapse observations is helpful for successful embryo transfer; however, information on the effect of male aging on time‐lapse is insufficient. We analyzed the impact of paternal aging on sperm characteristics, embryonic developmental kinetics, embryo evaluation score, and pregnancy outcomes.

**Methods:**

We used data from patients treated at our clinic between January 2020 and December 2022. We evaluated the effects of aging in men and women on semen data, in vitro fertilization (IVF) results, developmental kinetics, embryo evaluation scores, and embryo transfer outcomes using a retrospective approach.

**Results:**

Male aging adversely affected the semen characteristics. Although female aging had adverse effects on IVF, embryonic developmental kinetics, and embryo transfer outcomes, male aging did not have such a significant impact. Female aging decreased the iDAScore and Gardner criteria, whereas male aging did not affect the iDAScore.

**Conclusions:**

Aging in males had a negative effect on semen data. Contrary to the impact of aging on women, aging in men did not have a significant effect on embryo and gestation rates following embryo transfer.

## 1 INTRODUCTION

The decline in birth rate is a major social problem in developed countries. The background underlying the low birth rate is the increasing age of parents having their first child^1^ due to changes in plans in the lives of women and the poor economic status of couples. The most significant challenge for aged couples is age‐associated decline in fertility. Many studies have addressed age‐associated low fertility; however, most of them have focused on females. In this context, it has been shown that aging in women increases spindle dysplasia^2^, ^3^ and chromosomal abnormalities^4^, ^5^ and reduces mitochondrial quality^6^, ^7^, ^8^ in oocytes, which is believed to be a causal factor for low clinical pregnancy and high miscarriage rates^2^, ^9^ in aged women. However, the accumulation of empirical evidence has revealed that aging in men is a causal factor of low pregnancy rate^10^, ^11^; however, most studies have focused on basal information, such as sperm characteristics, rather than embryonic characteristics. A recent animal experiment using the same male mice showed that aging in males significantly affects mitochondrial numbers in embryos, and that differentially expressed genes are associated with mitochondrial functions.^12^, ^13^ From these results, it is plausible that embryos from aged fathers undergo certain metabolic changes that may affect their developmental kinetics.

A common strategy to achieve a high pregnancy rate in couples with low fertility is the selection of competent embryos. Embryonic grading has been conducted using morphological criteria, such as the Gardner criteria and Veeck classifications^14^, ^15^; however, these evaluations depend on simple noninvasive morphological information. Recently, time‐lapse incubators that allow the dynamic observation of embryonic development have become popular in assisted reproductive technology. Using a time‐lapse incubator, it has been shown that developmental kinetics are more closely associated with the quality of embryos and subsequent pregnancy outcome,^16^, ^17^, ^18^ and that female aging alters embryonic developmental kinetics.^16^ Furthermore, the IDAScore is a new artificial intelligence (AI)‐based numerical embryonic dataset obtained from a time‐lapse incubator system, Embryoscope (Vitrolife Vo Ltd.). Numerical data are easy to apply in embryonic evaluation, and female age has been reported to affect the accuracy of the iDAScore.^19^

In this study, we investigate the impact of paternal aging on sperm characteristics and evaluate the effects of paternal and maternal age on embryonic developmental kinetics, embryonic quality (as determined by the combined Gardner criteria or iDAScore), and pregnancy outcomes.

## 2 MATERIALS AND METHODS

### 2.1 Data origin and patient background

This study included 4240 cases of semen testing performed at our clinic between January 2020 and December 2022, during the period when all patients brought semen from their homes due to COVID‐19. Embryo analysis included 452 cases of conventional IVF [c‐IVF], 455 cases of intracytoplasmic sperm injection [ICSI], and 828 cases of frozen–thawed embryo transfer. In the analysis of fertilization rates and embryonic development, clinical cases in which no oocytes or more than 22 oocytes were collected were excluded from the database using the Smirnov–Grubbs test. Table 1 presents patient background data, including age and BMI, history of medical treatment (in our clinic), and the top five causal factors for infertility (diagnosis in our clinic). The history of pregnancy and abortion in each patient before visiting our clinic was not precisely disclosed; therefore, these data are not included in the table. The top causal factors in patients diagnosed in our clinic include male factor infertility, endometriosis, polycystic ovary syndrome (PCOS), recurrent abortion, and tubal factors, indicating a mixture of complex factors, which is a fundamental limitation in discussing the results in connection with certain infertility factors.

**TABLE 1:** Patient background.

Categories		SA^b^	IVF		ET
			c‐IVF	ICSI	
Total numbers		4240	452	455	828
Age	Male	36.1 ± 0.1	35.8 ± 0.2	40.6 ± 0.3	37.8 ± 0.2
	Female	34.5 ± 0.1	36.5 ± 0.2	38.1 ± 0.2	36.2 ± 0.1
BMI^a^	Male	23.1 ± 0.1	23.4 ± 0.2	23.2 ± 0.2	23.3 ± 0.1
	Female	34.5 ± 0.1	20.4 ± 0.1	20.7 ± 0.1	20.4 ± 0.1
History of medical treatment at our clinic	AIH (Times)	–	2.6 ± 0.1	1.9 ± 0.1	2.5 ± 0.1
	IVF (Times)	–	0.9 ± 0.1	1.7 ± 0.1	–
	ET (Times)	–	–	–	1.3 ± 0.1
Top 5 factors of infertility and percentage	Abbreviation	–			
	A: Unknown	–	A (61.2)	C (35.5)	A (43.6)
	B: Endometriosis	–	B (11.2)	A (34.1)	C (21.6)
	C: Male factor	–	C (8.5)	B (11.1)	B (10.6)
	D: PCOS	–	D (6.4)	E (6.4)	D (8.1)
	E: Recurrent abortion	–	E (5.8)	F (5.7)	E (5.5)
	F: Tubal factor	–			

*Note*: Data are presented as average ± standard error.

Abbreviations: BMI, Body Mass Index; ET, Embryo Transfer; IVF, In vitro fertilization including ICSI; PCOS, Polycystic ovary syndrome; SA, Semen Analysis.

^a^ BMI is calculated from height and weight at the initial visit.

^b^ Includes semen analysis for artificial insemination with husband and IVF.

### 2.2 Oocyte collection

All the patients were treated with an ovarian stimulation protocol using the clomiphene citrate stimulation, letrozole stimulation, or gonadotropin‐releasing hormone (GnRH) antagonist.^20^ The effects of ovarian stimulation were monitored using ultrasonography and hormonal profiles (estradiol [E_2_], luteinizing hormone, and progesterone). Ovulation triggering was performed with a GnRH agonist administered with a nasal spray formulation and human chorionic gonadotropin injection when the leading follicle reached 18 mm with a concomitant E_2_ level ≥ 250 pg/mL. Oocyte retrieval is usually performed 34–36 h after ovulation is triggered, and collected oocytes are incubated until insemination.

### 2.3 Semen analysis

All semen samples were brought from their homes because the experimental period overlapped with that of COVID‐19. Semen samples were liquefied at room temperature, and the MAKLER™ COUNTING CHAMBER was used to calculate semen volume (mL), sperm concentration, sperm motility rate (%), total sperm count, and abnormal sperm morphology. The *WHO manual for the examination and processing of human semen* [https://www.who.int/publications/i/item/9789240030787] was used to determine abnormal sperm morphology.

### 2.4 Insemination and embryo culture, frozen‐thawed transfer of embryo

Highly motile sperm cells were selected using density gradient centrifugation (Good Sperm) (NAKA Medical, Tokyo, Japan), followed by a swim‐up protocol for conventional in vitro fertilization (c‐IVF). Selected sperm cells were used for c‐IVF or intracytoplasmic sperm injection (ICSI). Using the standard c‐IVF protocol, oocytes were co‐incubated with 10 × 10^6^/mL spermatozoa in a fertilization medium for 5 h, and cumulus cells were removed from the oocytes by mechanical pipetting. For ICSI, oocytes were denuded by mechanical pipetting with 40 IU/mL hyaluronidase (Kitazato, Shizuoka, Japan) before ICSI. After insemination, the zygotes were placed individually in wells of equilibrated EmbryoSlide culture dishes (Vitrolife, Göteborg, Sweden) containing 25 μL of One Step Medium (ONESTEP) (NAKA Medical, Tokyo, Japan) and covered with Washed Oil (NAKA Medical, Tokyo, Japan). Incubation was conducted at 37°C under an atmospheric condition of 6% CO_2_, 5% O_2_, and 89% N_2_ in an EmbryoScope time‐lapse incubator (Vitrolife, Göteborg, Sweden). Expanded blastocysts were vitrified on day 5 or 6 after insemination using a vitrification media kit and Cryotop device (Kitazato, Shizuoka, Japan). Thawing procedures were performed using a thawing media kit (Kitazato, Shizuoka, Japan), and laser‐assisted hatching was performed (Laser; Saturn5™ active, CooperSurgical, USA). Frozen–thawed embryo transfers were conducted with endometrial preparations using either the natural ovulation cycle or hormone replacement therapy cycles. Embryo transfers were performed under transvaginal ultrasound guidance using an embryo transfer catheter (Kitazato, Shizuoka, Japan). Clinical pregnancy was defined as the presence of a gestational sac detected using ultrasonography at 2 weeks after embryo transfer.

### 2.5 Evaluation of in vitro fertilization and embryonic development

The effect of age on fertilization and embryonic development was evaluated using c‐IVF data. Fertilization was determined by the presence of two pronuclei.

### 2.6 Evaluation of developmental kinetics

Developmental kinetic analysis utilized data derived from ICSI, where the time point of sperm injection was identical. Images of individual blastocysts were retrospectively analyzed using Embryo Viewer equipped with an external computer workstation (Vitrolife, Göteborg, Sweden), and the timing of embryonic developmental events during culture from post‐insemination to the blastocyst stage was evaluated as described in a previous report.^21^ Morphokinetic parameters are pronuclear fading (singamy: tPNf), onset of 2–8 cell divisions (time of embryos to reach 2–8 cell stage: t2, t3, t4, t5, t6, t7, and t8), start of blastulation (first signs of a visible blastocoel: tSB), and complete blastocyst formation (just before ZP thinning: tB).

### 2.7 Evaluation of blastocysts

Before the analysis, the iDAScore of blastocysts derived from c‐IVF and those from ICSI were compared, and the average values were comparable between the two groups (4.7 vs. 4.5; detailed information is provided in Table S1). Therefore, all blastocysts derived from ICSI and c‐IVF were evaluated using the Gardner criteria and the iDAScore. The blastocysts were evaluated on days 5 and 6 after c‐IVF and ICSI, respectively. The iDAScore was developed using deep learning and a neural network trained to analyze time‐lapse sequence images of the embryos.^22^, ^23^ In the present study, we utilized iDAScore version 2.0, a fully automated AI model that rates embryos between 1.0 and 9.9 using an updated three‐dimensional convolution algorithm, considering both morphological and morphokinetic patterns determined by time‐lapse images.^24^ This numerical evaluation score is more helpful for embryonic evaluation than the iDA score Ver 1.0, which rates embryos from 7.0 to 9.9.^19^

### 2.8 Statistical analysis

Data were statistically analyzed using Bell Curve for Excel, version 4.05 (Social Survey Research Information Co., Ltd.). Correlations between male and female ages were analyzed using the Spearman rank‐order correlation coefficients (rs). Semen data, developmental kinetics, and blastocyst evaluation by the iDASCore were analyzed using the Kruskal–Wallis test, followed by the Steel–Dwass test for multiple comparisons. Fertilization and blastocyst rates and outcomes of the frozen–thawed embryo transfer were analyzed using the χ^2^ test. Residual analysis was performed when differences were observed. Values were considered statistically significant at *p* < 0.05.

## 3 RESULTS

### 3.1 Semen analysis

**FIGURE 1:**
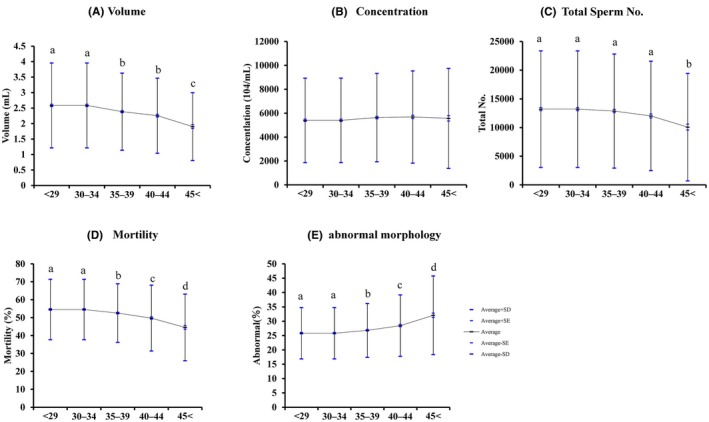
Effect of male age on general semen data. Effect of male aging on sperm characteristics: (A) Semen volume (mL). (B) Sperm concentration. (C) Total sperm number. (D) Sperm motility rates. (E) Abnormal sperm morphology. A–E: *p* < 0.05. Ber indicates standard deviation and standard error. X axis: Age of donor.

The results of the semen analysis are shown in Figure 1. Semen volume significantly decreased as men aged (*p* < 0.05), whereas sperm concentration did not change among male age groups (Figure 1A,B). Total sperm count and motility decreased as age increased in men, whereas abnormal morphology rates significantly increased as age increased (Figure 1C–E).

### 3.2 Age of patient and partner and male age groups

As the age of the couples correlated positively (*n* = 6880, rs = 0.65, *p* < 0.01, Figure S1), male age was divided into three groups based on the analysis of semen. Based on the results, the male age was divided into three groups: <34 years, 35–44 years, and >45 years.

### 3.3 Effect of age on IVF outcomes

The effects of aging on fertilization rates were evaluated using cases derived from c‐IVF and ICSI data separately. Table 2 shows that no age‐associated difference was found in females, but the fertilization rate for males aged 35–44 years was the highest in the 35–39‐year‐old female group (70.6%). When the same comparison was conducted using ICSI data, neither male nor female age‐associated decline in fertilization rate was found (Table 3). Furthermore, based on the data derived from c‐IVF, no age‐associated decline in the developmental rate of blastocysts was found for either males or females (Table 2); however, the developmental rate for women aged over 40 was higher than that for men aged under 45 (88.0%). Based on the ICSI data, female aging did not affect the developmental rate of blastocysts; however, in male aging, the rate was lowest for men aged 35–44 in the group with females aged over 40.

**TABLE 2:** Effect of age on fertilization and blastocyst rate with c‐IVF data.

partner Age	Gender examined	Age groups	Average age		No. of		Rate (%) of	
			Male	Female	Cycles	Oocytes	Fertil	Blast
>34	Male	<34	31.2	31.6	107	625	65.6	72.6
		35–44	37.6	32.8	62	349	71.1	76.9
		45<	47.3	33.6	3	37	62.1	62.1
35–39		<34	32.1	36.3	43	213	59.2	70.4
		35–44	38.1	36.9	149	725	70.6*	71.7
		45<	40.2	41.3	12	62	59.5	59.5
40<		<34	47.3	33.7	12	32	71.9	69.6
		35–44	47.0	37.3	51	163	72.4	73.0
		45<	47.2	40.9	13	38	88.0	88.0
<34	Female	<34	31.2	31.6	107	625	65.6	72.6
		35–39	37.6	32.8	43	213	59.2	70.4
		40<	47.3	33.7	12	32	69.6	69.6
35–44		<34	32.1	36.3	62	349	71.1	76.9
		35–39	38.1	36.9	149	725	70.6	71.7
		40<	40.2	41.3	51	163	73.0	73.0
45<		<34	41.9	32.8	3	37	78.4	62.1
		35–39	47.0	37.3	12	62	62.9	59.5
		40<	47.2	40.9	13	38	88.0	88.0*

*Note*: Women were divided into three groups (<34, 35–39, and >40 years) and data were divided into three male age groups (<34, 35–44, and >45 years). Men were divided into three groups (<34, 35–44, and >45 years) in which data were divided into three age groups (<34, 35–39, and >40 years).

Abbreviations: Blast, Blastocyst; Ferti, Fertilization.

* *p* < 0.05; The data was significantly higher or lower among the whole data (Residual analysis followed by χ^2^ test).

**TABLE 3:** Effect of age on fertilization and blastocyst rates with ICSI data.

Partner age	Gender examined	age Groups	Average age		No. of		Rate (%) of	
			Male	Female	Cycles	Oocytes	Fertil	Blast
>34	Male	<34	31.7	31.7	60	356	74.4	63.2
		35–44	38.5	32.2	37	180	81.7	72.7
		45<	45.9	33.6	7	45	62.9	62.9
35–39		<34	32.7	36.5	23	108	75.9	61.3
		35–44	38.7	37.0	117	452	77.9	66.2
		45<	47.8	37.4	29	143	70.5	70.5
40<		<34	33.1	43.9	18	55	83.6	81.8
		35–44	40.8	42.0	86	261	75.5	68.3*
		45<	51.9	43.2	78	192	72.9	72.9
<34	Female	<34	31.7	31.7	60	356	74.4	63.2
		35–39	32.7	36.5	23	108	75.9	61.3
		40<	33.1	43.9	18	55	81.8	81.8
35–44		<34	38.5	32.2	37	180	81.7	72.7
		35–39	38.7	37.0	117	452	77.9	66.2
		40<	40.8	42.0	86	261	68.3	68.3
45<		<34	45.9	33.6	7	45	82.2	62.9
		35–39	47.8	37.4	29	143	73.4	70.5
		40<	51.9	43.2	78	192	72.9	72.9

*Note*: Women were divided into three groups (<34, 35–39, and >40 years) and data were divided into three male age groups (<34, 35–44, and >45 years). Men were divided into three groups (<34, 35–44, and >45 years) in which data were divided into three age groups (<34, 35–39, and >40 years).

Abbreviations: Blast, Blastocyst; Ferti: Fertilization.

* *p* < 0.05; The data was significantly higher or lower among the whole data (Residual analysis followed by χ^2^ test).

### 3.4 Effect of age on embryogenesis kinetics

Table 4 shows the effects of female and male age on syngamy time (tPNf), time to reach the eight‐cell stage embryo (tPNf–t8), and full blastocyst arrival time (tPNf–tB). This analysis used data derived from ICSI because the time point of sperm injection was clearly defined. Male age did not affect the kinetics of embryonic development. However, female aging extended the time to reach the blastocyst stage and shortened the duration of syngamy in the 35‐ to 44‐year‐old male group.

**TABLE 4:** Effect of age on developmental kinetics.

Partner age	Gender examined	Age groups	No. of blastocysts	Developmental kinetics		
				tPNf	tPNf–t8	t8–tB
>34	Male	<34	119	22.0	34.9	47.1
		35–44	75	22.1	34.7	46.2
		45<	16	22.1	39.7	48.3
35–39		<34	41	22.9	33.2	50.3
		35–44	180	22.6	35.1	49.6
		45<	51	21.9	35.8	49.1
40<		<34	17	22.7	31.6	48.5
		35–44	84	21.4	34.5	50.5
		45<	55	22.6	34.4	52.7
<34	Female	<34	119	22.0	34.9	47.1
		35–39	41	22.9	33.2	50.3
		40<	17	22.7	31.6	48.1
35–44		<34	75	22.1ab	34.7	46.2a
		35–39	180	22.7a	35.1	49.6ab
		40<	84	21.4b	34.5	50.5b
45<		<34	16	22.1	39.7	48.3
		35–39	51	22.3	35.8	49.1
		40<	55	22.6	34.4	52.6

*Note*: The time points of each event were represented as hours after sperm injection. a,b: *p* < 0.05.

Abbreviations: tPNf, Time of syngamy; tPNf‐t8, Time from tPNf to eight cells; t8–tB, Time from eight cells to just before blastocyst expansion.

### 3.5 Effect of age on the outcome of frozen–thawed embryo transfer

The pregnancy and abortion rates are presented in Table 5. For this comparison, data from IVF and ICSI were merged. Male aging did not affect the outcome of embryo transfer, whereas the effect of female aging was prominent, with a significant age‐associated reduction in the GS rate for women >40 in the <34 and 34–44 male groups (Table 5).

**TABLE 5:** Effect of age on the outcome of frozen–thawed embryo transfer.

Partner age	Ave. of partner age	Gender examined	Age groups	No. of cycles	Gs+ rate (%)	Abortion rate (%)	iDAScore
>34	31.5	Male	<34	164	58.5	18.8	6.0
	32.6		35–44	111	64.0	16.9	6.6
	33.7		45<	10	50.0	0.0	5.6
35–39	36.4		<34	54	57.4	41.9	6.4
	36.9		35–44	268	48.1	25.6	5.7
	37.2		45<	45	40.0	16.7	5.8
40<	42.4		<34	21	23.8	40.0	5.3
	41.5		35–44	106	30.2	40.6	5.2
	42.0		45<	49	28.6	50.0	5.2
<34	31.2	Female	<34	164	58.5	18.8	6.0
	32.5		35–39	54	57.4	41.9	6.4
	32.9		40<	21	23.8*	40.0	5.3
35–44	38.2		<34	111	64.0*	16.9	6.6a
	38.5		35–39	268	48.1	25.6	5.7b
	39.5		40<	106	30.2*	40.6	5.4b
45<	46.4		<34	10	50.0	0.0	5.6
	48.4		35–39	45	40.0	16.7	5.8
	48.9		40<	49	28.6	50.0	5.2

*Note*: The abortion rate was calculated as the number of abortions per clinical pregnancy. Embryo evaluation was performed retrospectively using simultaneous iDAScore Ver. 2 calculations of data within the study period.

* *p* < 0.05; The data were significantly higher or lower compared to the whole data (Residual analysis followed by a χ^2^ test).

### 3.6 Effect of age on blastocyst evaluation using iDAScore Ver.2

Blastocysts derived from the male and female age groups were evaluated using iDAScore ver. 2 (Table 5).

**FIGURE 2:**
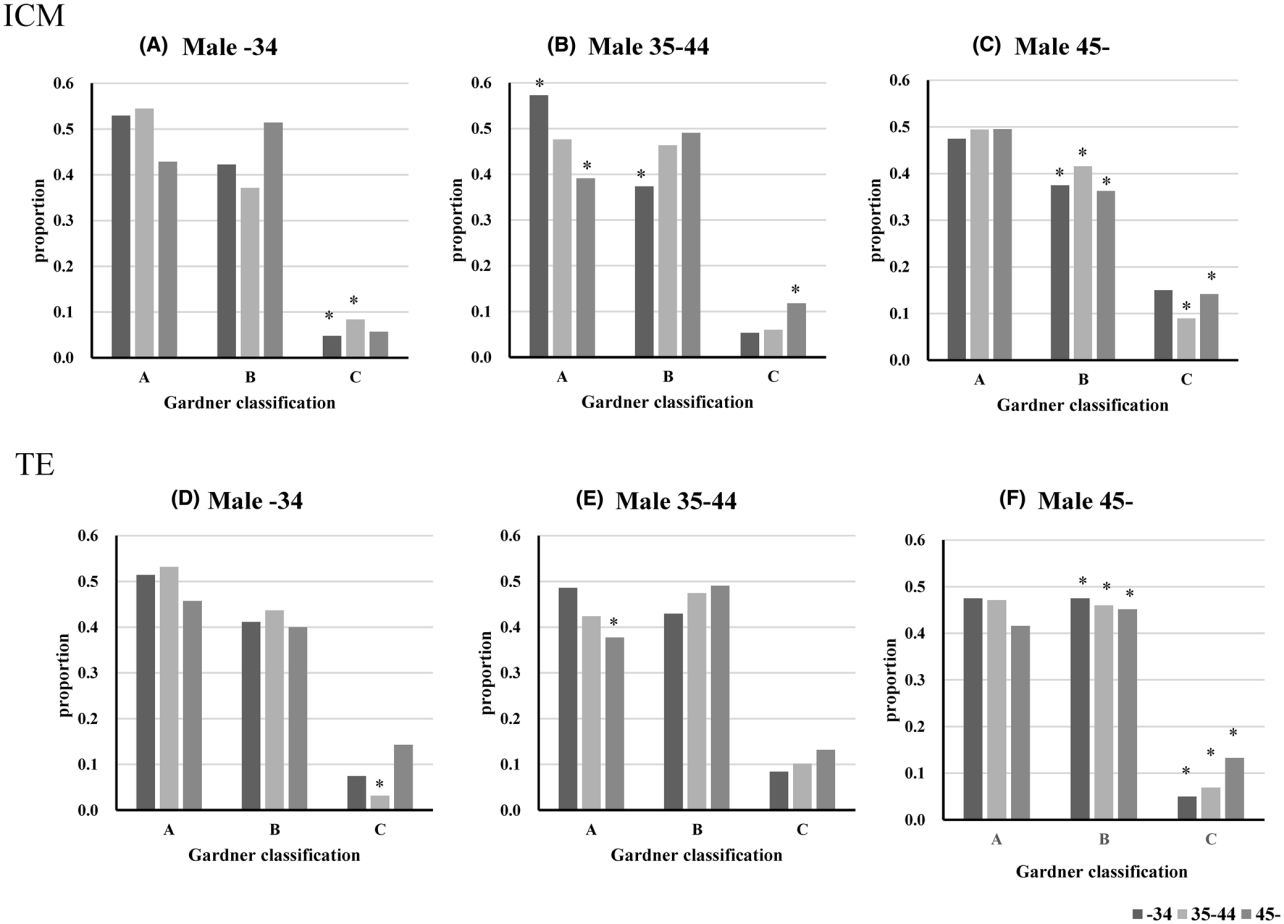
Effect of female age on blastocyst evaluation using Gardner's criteria. Embryos were evaluated using Gardner's criteria. *p < 0.05; The data were significantly higher or lower compared to the whole data, as evaluated by residual analysis following the χ^2^ test.

**FIGURE 3:**
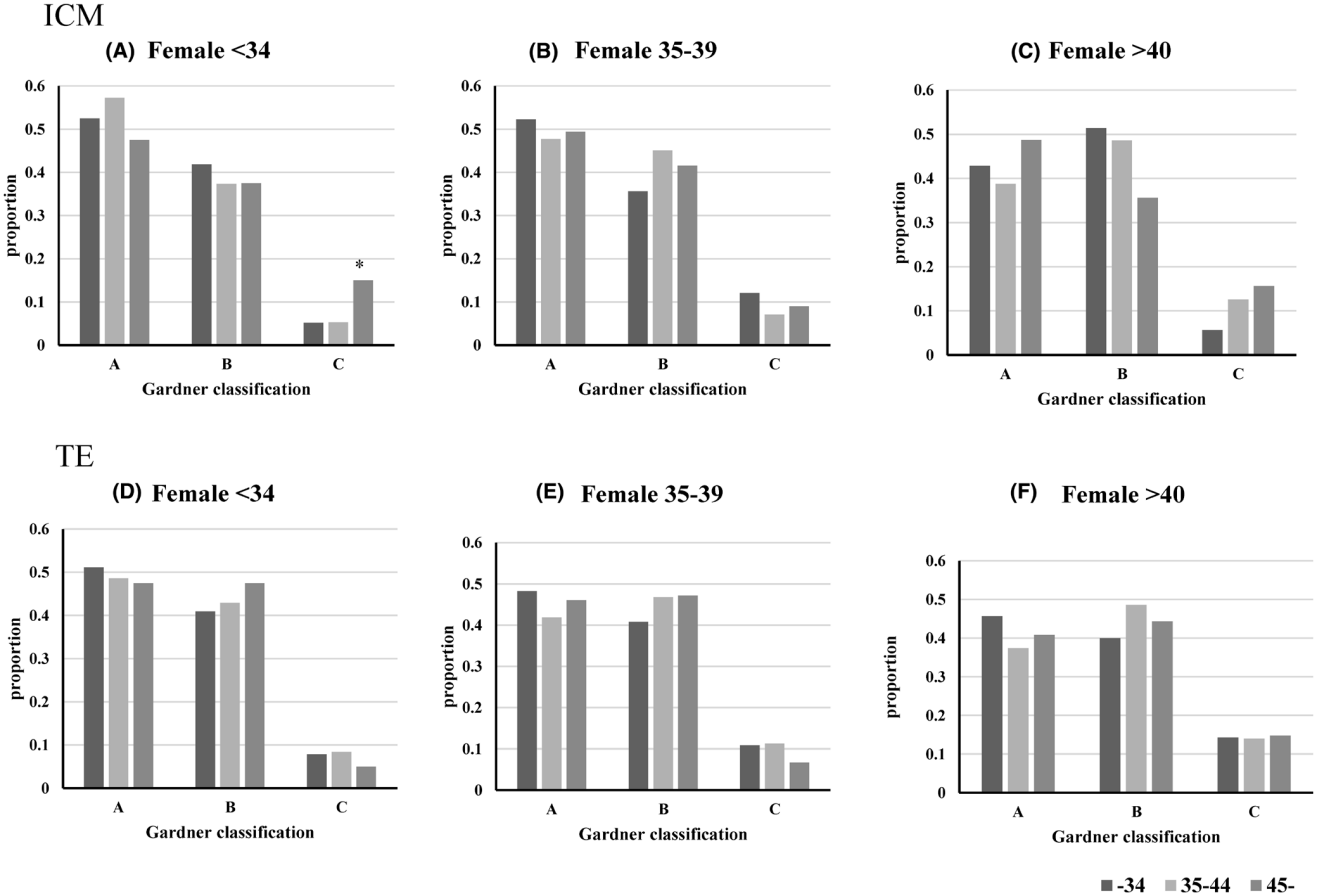
Effect of male age on blastocyst evaluation using Gardner's criteria. Embryos were evaluated using Gardner's method. *p < 0.05; The data were significantly higher or lower among the whole data that were evaluated by residual analysis following a χ^2^ test.

In all three female age groups, no significant effect of male age on the iDAScore was observed. In the 35–44 male group, the iDAScore of the <34 years female age group was significantly higher than that of the 35–44 and >45‐year‐old groups. When the identical embryos were evaluated using the conventional Gardner criteria, the results were presented in Figure 2A–F. Age‐dependent decrease and increase in the proportion of A and C grad were observed in the inner cell mass (ICM) of the male 35–44 groups (Figure 2B), age‐dependent decrease and increase in the proportion of B and C grade were observed in the trophectoderm (TE) of the male >45 group (Figure 2F). Consistent with the iDAcore, male age‐associated changes in Gardner classification are small (Figure 3A–F), but an age‐associated increase in the proportion of C grad was observed in the ICM of the female <34 group (Figure 3A).

## 4 DISCUSSION

The present study showed that paternal aging increases the proportion of sperm with abnormal morphology and decreases seminal volume, concentration, and motility. Additionally, maternal aging has a profound impact on embryonic development and kinetics, as well as gestation rate and embryonic evaluation scores, whereas paternal aging does not affect embryo development, kinetics, or gestation rate, despite using the same database. Additionally, the embryonic evaluation score (iDAScore) was ineffective in detecting paternal aging‐associated changes.

### 4.1 Patient background and semen analysis

The age of the partners was significantly correlated. Several reports have shown that maternal aging negatively affects oocyte quality^25^, ^26^, ^27^ and IVF outcomes.^2^, ^7^, ^27^ Therefore, a careful evaluation of the effects of paternal aging on embryonic development and IVF outcomes is required. In the present study, a decline in semen parameters was observed from the age of 35 and became more pronounced after 45 in males. Additionally, the proportion of abnormal seminal morphology increased after the age of 45. The age‐associated decline in semen parameters is consistent with a report^28^ that showed that semen motility, morphology, and volume decreased in males in their 50s compared to their counterparts in their 30s. Furthermore, Ezoe et al. reported that semen motility declined in males aged >42 years.^29^ Based on these results, we divided the male age group into three categories: <35, 35–44, and >44 years. The location of semen collection limited the semen analysis, as the experimental period overlapped with the COVID‐19 pandemic, and the transportation effect on semen could not be excluded.

### 4.2 Effect of female aging on embryos

Several studies have been published regarding the age‐associated decline in the oocyte's ability to fertilize and develop to term.^30^, ^31^ In the present study, we observed that the age‐associated decline in gestation rate, embryonic score, and slow embryonic development, as well as the developmental rate to the blastocyst stage, was comparable among the three female age groups. However, a very high developmental rate to the blastocyst stage was observed in both the male group (>45%) and the female group (>40%) (88%). The causal factor behind this high rate was unclear, but the small sample size and patient‐specific background may have contributed to this exception. Slow and low embryonic development has been previously reported. For example, Rose et al. reported that embryonic development to the blastocyst stage decreased with increasing age.^32^ Ezoe et al. reported that aging in women slowed embryonic development and decreased blastocyst arrival rates,^29^ and Ueno et al. reported a decrease in embryo quality. Moreover, the developmental rate of the blastocyst stage remained stable until the age of 40 years, with a notable reduction in women aged 41 years and older.^33^ Aging in women increases the rate of embryonic aneuploidy,^2^, ^4^, ^34^ leading to slower embryonic development.^35^ However, many studies have reported a low impact of maternal aging on fertilization. Yan et al. reported that the normal fertilization rate and number of good embryos did not change with age in women.^36^ Uzun et al. reported no difference in either the normal fertilization rate or the rates of 1 PN or ≥3 PN between women aged under 35 and those aged 35 years or older.^37^ Furthermore, Romanski reported that the normal fertilization rate in IVF remained relatively stable until the age of 44 years, with >80% of the oocytes being successfully fertilized.^33^ Thus, maternal aging decreases the embryonic developmental ability of oocytes, but this difference is difficult to detect in the first‐stage zygotes. Notably, we showed for the first time that the timing of pronuclear disappearance is faster in the embryos of aged women. We previously reported that pronuclear formation was faster in embryos of aged cows because of the rapid decline in MPF activity.^38^ Therefore, we suggest that a similar mechanism may underlie the disappearance of the first PN in the oocytes of aged women. It is important to detect low‐quality embryos using noninvasive methods. Based on the results of the present study, slow embryonic developmental kinetics is a good indicator of aging, and the phenotype can be easily detected using time‐lapse incubators. Furthermore, the present study showed that both the iDAScore and the Gardner criteria reflected a female age‐associated decline in embryo quality in the same male age groups; however, the Gardner criteria appear to be more sensitive. The iDAScore is an AI‐assisted embryonic evaluation method that utilizes embryonic developmental kinetics and morphology, with numerical values that can be easily applied in any clinic. Thus, time‐lapse incubators and embryonic scores are useful for detecting age‐associated decline in female embryo quality.

### 4.3 Effect of male aging on embryos

The effects of male aging on embryonic quality have attracted the attention of several researchers. In a previous study involving 221 couples, male aging was associated with a reduced pregnancy rate.^39^ In contrast, a report of 9991 cases, in which data were divided into three groups using the age of couples (<30, 30–34, and 35–38), found no male aging‐associated decline in fertilization, implantation, or pregnancy rates.^40^ In the present study, embryonic development, developmental kinetics, embryonic evaluation scores, and the outcome of embryo transfer were comparable among the three male age groups. One exception is the differences in fertilization rates in the 35–39 female category, where the rate in the <34 and >45 male groups was relatively low compared with the other >34 and >40 female categories. The background of this difference was unclear and required more detailed study. Animal studies in mice have reported that male aging reduces embryonic developmental ability.^35^ In addition, aging of C57Bl/6J male mice reduced sperm parameters and deteriorated embryonic developmental ability in blastocysts.^41^ In contrast, aging of C57B/6 male mice reduced the mitochondrial DNA copy number in blastocysts and affected the expression levels of genes associated with mitochondria in blastocysts; however, these authors did not detect differential embryonic developmental ability.^12^, ^13^ In conjunction with these reports, it has been suggested that male aging leads to the deterioration of embryonic characteristics, whereas age‐associated changes are not necessarily perceived as adverse embryonic phenotypes. Contrary to this hypothesis, contradictory reports have been published on the effects of aging on pregnancy outcomes in men. Aging in men adversely affects pregnancy outcomes and abortion rates,^11^, ^34^, ^41^ but Begueria et al. reported that the age of men only affects semen parameters but does not affect embryonic quality and pregnancy outcome following embryo transfer.^42^ Accumulating evidence has shown that paternal aging decreases IVF outcomes and increases the risk to the child.^11^, ^41^, ^43^ These cases require noninvasive embryonic evaluation, which detects age‐associated changes in embryos. In the present study, we evaluated two noninvasive methods to determine whether these parameters reflect paternal aging: embryonic developmental kinetics, which can be easily obtained using time‐lapse incubators, and numerical embryonic scores, which are also automatically obtained from the time‐lapse dataset. In contrast to the effect of female aging, embryonic developmental kinetics did not differ among male age groups. Furthermore, the iDAScore did not detect differences between embryos derived from different male age groups. However, the Gardner criteria showed slight but significant age‐associated differences, indicating that although the Gardner criteria are complex and subjective, well‐trained operators can detect certain changes in embryos derived from aged men. However, there is currently no sensitive empirical method for detecting abnormal embryonic phenotypes. Further studies are required to develop or improve the current evaluation methods. A limitation of the present study was its small sample size, which hindered better comparison within each age group. We prepared 907 case data points for analysis; however, to elicit the intrinsic paternal aging effect, the data should be divided into female age groups. Therefore, larger volumes of data are required to reach clear conclusions.

## 5 CONCLUSION

Male aging has a negative impact on semen characteristics, including decreased semen volume, total sperm count, and motility, as well as increased abnormal morphology. In contrast to the significant impact of female aging on embryonic development, male aging had no notable effects. Time‐lapse incubators provide useful data, such as embryonic developmental kinetics and embryo evaluation scores, which reflect changes in embryos associated with female aging but not male aging.

## CONFLICT OF INTEREST STATEMENT

The authors declare no Conflict of Interest for this article. Hisataka Iwata is an Editorial Board member of Reproductive Medicine and Biology and a co‐author of this article. To minimize bias, he was excluded from all editorial decision‐making related to the acceptance of this article for publication.

## ETHICS STATEMENT

The Institutional Review Board of the Mine Ladies Clinic approved the study design (Approval no. 2;2023). Informed consent was obtained from all couples who were informed that their unidentified data could be used for retrospective analyses.

## CONSENT

Informed consent was obtained from all the patients included in the study.

## HUMAN RIGHTS STATEMENTS

All procedures were performed in accordance with the ethical standards of the relevant committees on human experimentation (institutional and national) and the Declaration of Helsinki of 1964 and its later amendments.

## ANIMAL RIGHTS

This report does not contain any animal studies performed by any of the authors.

## Supporting information


Data S1.

## Data Availability

All datasets will be provided by the corresponding author upon reasonable request.
