# Supplementary material for: Effect of aging on semen and embryonic developmental scores in assisted reproductive technology
Source: Reprod Med Biol. 2025 May 21;24(1):e12647. doi: 10.1002/rmb2.12647 (PMC12094255; doi:10.1002/rmb2.12647)
Supplement: Supplementary file 1 — Data S1. [file RMB2-24-e12647-s001.docx]

Supplementary Figure 1


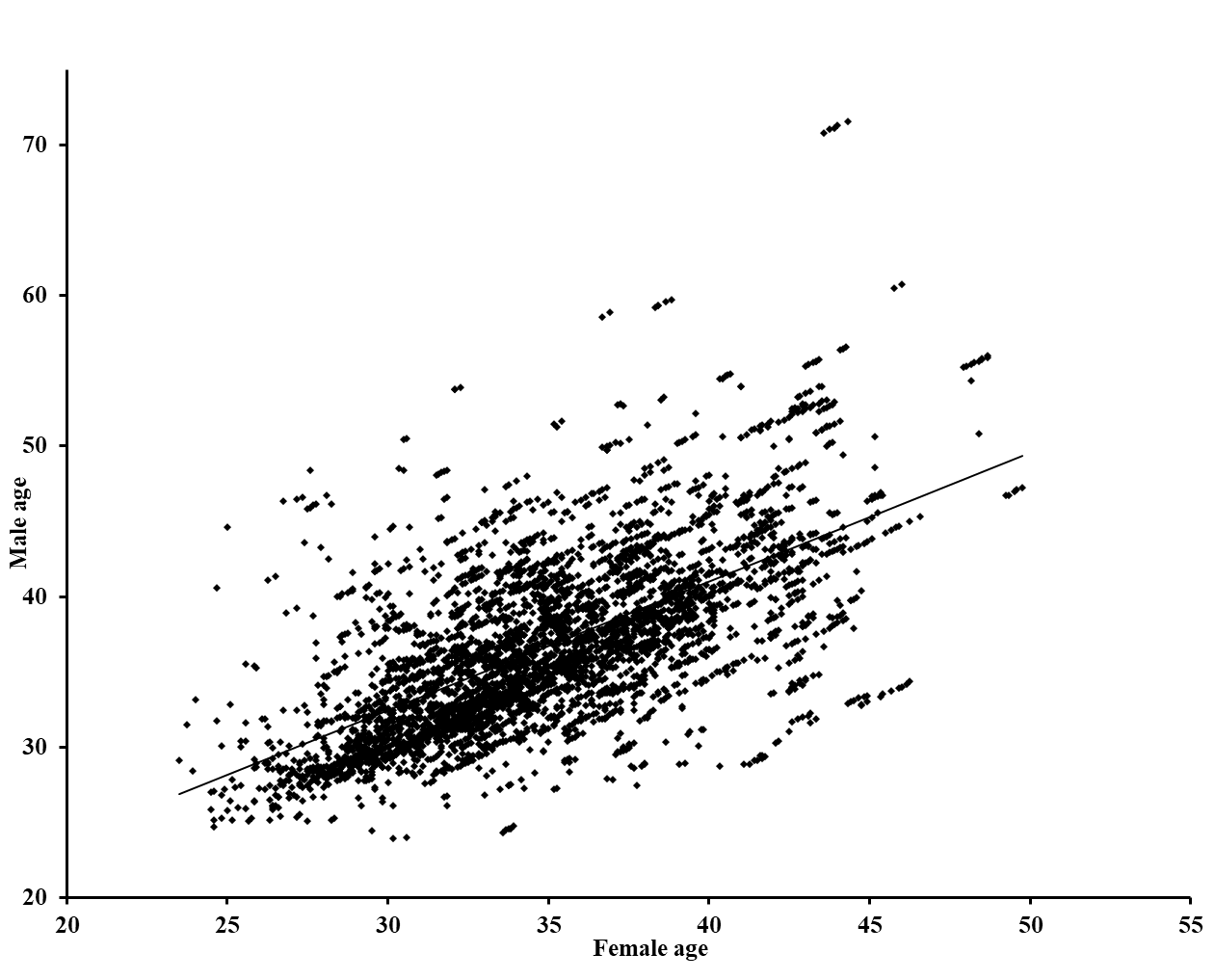


S-Fig. 1 Age distribution of couples at the time of sperm collection

Multiple sperm collections from the same couple were counted. A positive correlation was found between the ages of the men and women (n=4,240, rs=0.65, P < 0.01).

Supplementary Table 1.

Before merging c-IVF and ICSI data, embryonic evaluation score was compared between the two origins. Although the patients age was different, embryonic score was comparative.
